# Struggle To Survive: the Choir of Target Alteration, Hydrolyzing Enzyme, and Plasmid Expression as a Novel Aztreonam-Avibactam Resistance Mechanism

**DOI:** 10.1128/mSystems.00821-20

**Published:** 2020-11-03

**Authors:** Ke Ma, Yu Feng, Alan McNally, Zhiyong Zong

**Affiliations:** a Center of Infectious Diseases, West China Hospital, Sichuan University, Chengdu, Sichuan, China; b Division of Infectious Diseases, State Key Laboratory of Biotherapy, Chengdu, Sichuan, China; c Center for Pathogen Research, West China Hospital, Sichuan University, Chengdu, Sichuan, China; d Institute of Microbiology and Infection, College of Medical and Dental Sciences, University of Birmingham, Birmingham, United Kingdom; e Department of Infection Control, West China Hospital, Sichuan University, Chengdu, Sichuan, China; University of Illinois at Chicago

**Keywords:** aztreonam-avibactam, avibactam, CMY, penicillin-binding protein, plasmid copy number, *Escherichia coli*, antibiotic resistance, aztreonam

## Abstract

Aztreonam-avibactam is a promising antimicrobial combination against multidrug-resistant organisms, such as carbapenemase-producing *Enterobacterales*. Resistance to aztreonam-avibactam has been found, but the resistance mechanism remains poorly studied. We recovered three Escherichia coli isolates of an almost identical genome but exhibiting varied aztreonam-avibactam resistance. The isolates carried a cephalosporinase gene, *bla*_CMY-42_, on IncIγ plasmids with a single-nucleotide variation in an antisense RNA-encoding gene, *inc*, of the replicon. The isolates also had four extra amino acids (YRIK) in penicillin-binding protein 3 (PBP3) due to a duplication of a 12-nucleotide (TATCGAATTAAC) stretch in *pbp3*. By cloning and plasmid-curing experiments, we found that elevated CMY-42 cephalosporinase production or amino acid insertions in PBP3 alone mediated slightly reduced susceptibility to aztreonam-avibactam, but their combination conferred aztreonam-avibactam resistance. We show that the elevated CMY-42 production results from increased plasmid copy numbers due to mutations in *inc*. We also verified the findings using *in vitro* mutation assays, in which aztreonam-avibactam-resistant mutants also had mutations in *inc* and elevated CMY-42 production compared with the parental strain. This choir of target modification, hydrolyzing enzyme, and plasmid expression represents a novel, coordinated, complex antimicrobial resistance mechanism and also reflects the struggle of bacteria to survive under selection pressure imposed by antimicrobial agents.

**IMPORTANCE** Carbapenemase-producing *Enterobacterales* (CPE) is a serious global challenge with limited therapeutic options. Aztreonam-avibactam is a promising antimicrobial combination with activity against CPE producing serine-based carbapenemases and metallo-β-lactamases and has the potential to be a major option for combatting CPE. Aztreonam-avibactam resistance has been found, but resistance mechanisms remain largely unknown. Understanding resistance mechanisms is essential for optimizing treatment and developing alternative therapies. Here, we found that either penicillin-binding protein 3 modification or the elevated expression of cephalosporinase CMY-42 due to increased plasmid copy numbers does not confer resistance to aztreonam-avibactam, but their combination does. We demonstrate that increased plasmid copy numbers result from mutations in antisense RNA-encoding *inc* of the IncIγ replicon. The findings reveal that antimicrobial resistance may be due to concerted combinatorial effects of target alteration, hydrolyzing enzyme, and plasmid expression and also highlight that resistance to any antimicrobial combination will inevitably emerge.

## Abstract (precis)

Carbapenemase-producing *Enterobacterales* (CPE) is a serious global challenge with limited therapeutic options. Aztreonam-avibactam is a promising antimicrobial combination with activity against CPE producing serine-based carbapenemases and metallo-β-lactamases and has the potential to be a major option for combatting CPE. Aztreonam-avibactam resistance has been found, but resistance mechanisms remain largely unknown. Understanding resistance mechanisms is essential for optimizing treatment and developing alternative therapies. Here, we found that either penicillin-binding protein 3 modification or the elevated expression of cephalosporinase CMY-42 due to increased plasmid copy numbers does not confer resistance to aztreonam-avibactam, but their combination does. We demonstrate that increased plasmid copy numbers result from mutations in antisense RNA-encoding *inc* of the IncIγ replicon. The findings reveal that antimicrobial resistance may be due to concerted combinatorial effects of target alteration, hydrolyzing enzyme, and plasmid expression and also highlight that resistance to any antimicrobial combination will inevitably emerge.

## INTRODUCTION

The ongoing rise in the prevalence of multidrug-resistant (MDR) bacterial pathogens has led to a global concerted effort to combat this most serious of global health threats (1). The rise in antimicrobial resistance is an extremely complex and multifactorial problem (2). Studying the mechanisms responsible for resistance to antimicrobial agents of clinical significance in common bacterial pathogens generates critically important insights for combating antimicrobial resistance (1, 3). The *Enterobacterales* is an order of Gram-negative bacteria, such as Escherichia coli, Klebsiella pneumoniae, and *Enterobacter* spp., that are widely distributed in nature and are major human pathogens, causing infections ranging from intestinal disease and urinary tract infections to invasive bloodstream infections and meningitis (4). Carbapenems such as ertapenem, imipenem, and meropenem are potent antimicrobial agents and the mainstream agents of choice to treat severe infections caused by the *Enterobacterales.* However, carbapenemase-producing *Enterobacterales* (CPE) has emerged worldwide, representing a serious challenge for clinical management and public health (5). Carbapenem resistance in *Enterobacterales* is mainly due to the production of carbapenem-hydrolyzing enzymes (carbapenemases) (6). Carbapenemases can be divided into two major types, i.e., serine-based enzymes with a serine residue in the active site, such as KPC (Klebsiella pneumoniae carbapenemase), and metallo-β-lactamases (MBLs) containing zinc in the active site, such as NDM (New Delhi metallo-β-lactamase) (6, 7).

Avibactam (AVI) is a recently developed non-β-lactam β-lactamase inhibitor with the ability to inhibit serine-based carbapenemases, but it cannot inhibit MBLs (8). The combination of ceftazidime-avibactam (CAZ-AVI) has been in clinical use but has no activity against MBL producers (9). Aztreonam (ATM) is stable to the hydrolysis of MBLs (10, 11), and the combination of aztreonam-avibactam (ATM-AVI) has activity against CPE producing serine-based carbapenemases, MBLs, or both and has the potential to be a major option for combatting CPE. Unfortunately, ATM-AVI-resistant strains have been found (12–14), but the resistance mechanisms remain poorly studied. In this study, we report a unique combination of ATM-AVI resistance mechanisms in Escherichia coli.

## RESULTS

### CMY-42 confers slightly reduced susceptibility to ATM-AVI.

E. coli isolates 035123, 035125, and 035148 were recovered from hospital sewage and were resistant to ATM-AVI at different levels (MIC, 16/4, 64/4, and 128/4 mg/liter, respectively) (Table 1; see also Table S1 in the supplemental material). We performed whole-genome sequencing for the three isolates. They belonged to a common strain with only one or two single-nucleotide polymorphisms (SNPs), and all had a 34,321-bp IncIγ plasmid carrying an AmpC-type cephalosporinase gene, *bla*_CMY-42_.

**TABLE 1:** MICs of antimicrobial agents against strains containing cloning fragments or plasmids

Strain and/or plasmid	MIC (mg/liter) of^*a*^:						Note
	ATM	ATM-AVI	ATM-AVI	CAZ	CAZ-AVI	CAZ-AVI	
035125	**128**	**16/4**	1/8	**1,024**	8/4	0.5/8	ST410, YRIK insertion in PBP3, carrying *bla*_CMY-42_, from sewage
035123	**512**	**64/4**	4/8	**>1,024**	**16/4**	1/8	ST410, YRIK insertion in PBP3, carrying *bla*_CMY-42_, from sewage
035148	**512**	**128/4**	**16/8**	**>1,024**	**32/4**	4/8	ST410, YRIK insertion in PBP3, carrying *bla*_CMY-42_, from sewage
005008	**>512**	4/4	0.5/8	**>1,024**	**64/4**	**16/8**	ST3835, YRIN insertion in PBP3, carrying *bla*_CMY-42_ and *bla*_NDM-1_, from ascites (17)
005008R1	**>512**	**64/4**	4/8	**>1,024**	**256/4**	**32/8**	ATM-AVI-resistant mutant of 050008, SNP in *inc*
005008R2	**>512**	**64/4**	4/8	**>1,024**	**256/4**	**32/8**	ATM-AVI-resistant mutant of 050008, SNP in *inc*
020066	**512**	8/4	1/8	**>1,024**	**64/4**	**16/8**	ST6823, YRIP insertion in PBP3, no *bla*_CMY-42_, carrying *bla*_NDM-1_, from urine (22)
BL21	0.015	0.015/4		0.015	0.015/4		
BL21:pET28a	0.015	0.015/4		0.015	0.015/4		
BL21::pET28a_CMY2	**32**	0.015/4		**32**	0.015/4		*bla*_CMY-2_ cloned on pET28a
BL21::pET28a_CMY42	**512**	0.03/4		**>1,024**	0.03/4		*bla*_CMY-42_ cloned on pET28a
035125ΔpCMY42	2	1/4		4	1/4		035125 without pCMY42_035125, resulting from plasmid curation
035125ΔpCMY42::pET28a	2	1/4		4	1/4		
035125ΔpCMY42::pET28a_CMY2	**32**	4/4	0.125/8	**32**	2/4	0.06/8	*bla*_CMY-2_ cloned on pET28a
035125ΔpCMY42::pET28a_CMY42	**512**	**64/4**	2/8	**>1,024**	**32/4**	1/8	*bla*_CMY-42_ cloned on pET28a
035125ΔpCMY42::pCMY42_035125	**128**	**16/4**	1/8	**1,024**	8/4	0.5/8	pCMY42_035125 introduced back into 035125ΔpCMY42
035125ΔpCMY42::pCMY42_035123	**512**	**32/4**	4/8	**>1,024**	**16/4**	1/8	pCMY42_035123 introduced into 035125ΔpCMY42
035125ΔpCMY42::pCMY42_035148	**1,024**	**128/4**	**16/8**	**>1,024**	**32/4**	4/8	pCMY42_035148 introduced into 035125ΔpCMY42
035125ΔpCMY42::pCMY42_005008	**128**	4/4	0.5/8	**1,024**	8/4	0.25/8	pCMY42_005008 introduced into 035125ΔpCMY42
035125ΔpCMY42::pCMY42_005008R1	**512**	**64/4**	2/8	**>1,024**	**64/4**	1/8	pCMY42_005008R1 introduced into 035125ΔpCMY42
035125ΔpCMY42::pCMY42_005008R2	**512**	**64/4**	2/8	**>1,024**	**64/4**	1/8	pCMY42_005008R2 introduced into 035125ΔpCMY42
BL21::pBC SK	0.015	0.015/4		0.015	0.015/4		
BL21:: *pbp3*_BL21	0.015	0.015/4		0.015	0.015/4		*pbp3* of BL21 cloned on pBC SK
BL21:: *pbp3*_YRIN	0.25	0.25/4		0.25	0.25/4		*pbp3* of 035125 cloned on pBC SK
BL21:: *pbp3*_YRIK	0.25	0.25/4		0.25	0.25/4		*pbp3* of 005008 cloned on pBC SK
BL21:: *pbp3*_YRIP	0.25	0.25/4		0.25	0.25/4		*pbp3* of 020066 cloned on pBC SK

a Resistance is highlighted in boldface.

10.1128/mSystems.00821-20.1 TABLE S1 MICs (mg/liter) of antimicrobial agents against strains 035123, 035125, 035148, 005008, and 005008R1. Download Table S1, DOCX file, 0.02 MB. Copyright © 2020 Ma et al. 2020 Ma et al. This content is distributed under the terms of the Creative Commons Attribution 4.0 International license.

CMY-42 has an amino acid substitution (Ser231Val at Ambler’s position 211) compared to CMY-2 (15). Protein structures of CMY-2 and CMY-42 were predicted (Fig. S1). Molecular modeling of both enzymes showed that compared with CMY-2, CMY-42 has a neonatal polar bond of 2.6 Å present between the hydrogen atom of 231Ser and oxygen atom of 232Ser and has an additional van der Waals force of 2.3 Å among atoms of 231Ser and the spatially adjacent amino acid residues.

10.1128/mSystems.00821-20.2 FIG S1 Protein structure of CMY-2 and CMY-42. The structure was predicted by I-TASSER and is visualized by PyMOL. (A) CMY-2; (B) CMY-42. Download FIG S1, PDF file, 0.7 MB. Copyright © 2020 Ma et al. 2020 Ma et al. This content is distributed under the terms of the Creative Commons Attribution 4.0 International license.

To examine whether CMY-42 confers ATM-AVI resistance, we cloned *bla*_CMY-42_ onto pET28a to construct pET28a_CMY42, which was introduced into strain BL21. We also cloned *bla*_CMY-2_ onto pET28a to construct pET28a_CMY2 as a control. The ATM MIC against BL21::pET28a_CMY42 was 512 mg/liter, 8-fold higher than that for BL21::pET28a_CMY2 (Table 1). It is evident that CMY-42 has significantly stronger activity on ATM than CMY-2. However, the presence of *bla*_CMY-42_ only slightly increased the ATM-AVI MIC from 0.015/4 mg/liter for BL21::pET28a to 0.03/4 mg/liter for BL21::pET28a_CMY42, while the presence of *bla*_CMY-2_ did not change the ATM-AVI MIC (0.015/4 mg/liter) (Table 1). This suggests that CMY-42 only slightly reduced susceptibility to the combined ATM-AVI. Of note, CMY-42 also confers slightly reduced susceptibility to CAZ-AVI (Table 1 and Table S1).

### PBP3 with the insertion of the four extra amino acids confers reduced susceptibility but not resistance to ATM-AVI.

**FIG 1:**
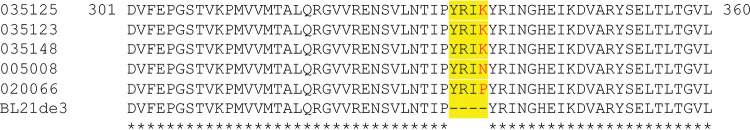
Alignment of the insertion region of the *pbp3* gene. The alignment was generated using Clustal Omega (42). The four extra amino acid insertions are highlighted in yellow. A complete alignment of the *pbp3* gene sequence is shown in Fig. S2.

The insertion of amino acids in penicillin-binding protein 3 (PBP3), due to duplication of nucleotide sequence, has been found to reduce susceptibility to ATM-AVI (13, 16). All three isolates had a duplication of a 12-nucleotide sequence (TATCGAATAAC) in *pbp3*, resulting in four extra amino acids (YRIK) in PBP3 (Fig. 1 and Fig. S2). We cloned *pbp3* of 035125 (*pbp3*_YRIK) into BL21 and also cloned *pbp3* of BL21 (*pbp3*_BL21) as a control. The MIC of ATM-AVI for BL21::*pbp3*_YRIK was 0.25/4 mg/liter, 16-fold the MIC of 0.015/4 mg/liter for BL21::*pbp3*_BL21 (Table 1). This confirms that the YRIK insertion in PBP3 reduces susceptibility to ATM-AVI but at a level below the resistance breakpoint. However, the recipient strain BL21 has its own *pbp3* gene (*pbp3_*BL21), which has no duplication of nucleotide sequence but may blur the effect of *pbp3*_YRIK. We then cured the *bla*_CMY-42_-carrying plasmid pCMY42_035125 from 035125 using SDS to remove the additive effect provided by CMY-42. The absence of pCMY42_035125 from 035125ΔpCMY42 was confirmed by PCR. The MIC of ATM and ATM-AVI against 035125ΔpCMY42 was 2 and 1/4 mg/liter (Table 1), respectively. This confirms that the YRIK insertion in PBP3 is unable to confer resistance to ATM-AVI but confers reduced susceptibility. In addition, the YRIK insertion in PBP3 has the same impact on CAZ-AVI (Table 1).

10.1128/mSystems.00821-20.3 FIG S2 Alignment of *pbp3* gene sequences. The alignment was generated using Clustal Omega. The four extra amino acid insertions are highlighted in yellow. Download FIG S2, PDF file, 0.02 MB. Copyright © 2020 Ma et al. 2020 Ma et al. This content is distributed under the terms of the Creative Commons Attribution 4.0 International license.

A previous study also reported another type of four-amino-acid insertion, YRIN, in PBP3 of E. coli (13). We also found such YRIN insertion in PBP3 of E. coli strain 005008 (17) and detected another type of four-amino-acid insertion, YRIP, in PBP3 of E. coli strain 020066 (18) in our collections. To examine the impact of the YRIN and YRIP insertions in PBP3, we cloned *pbp3* of 005008 (*pbp3*_YRIN) and that of 020066 (*pbp3*_YRIP) into BL21. The ATM-AVI MIC for both BL21::*pbp3*_YRIN and BL21::*pbp3*_YRIP was 0.25/4 mg/liter, the same as that for BL21::*pbp3*_YRIK (Table 1). This suggests that insertions of YRIN, YRIK, and YRIP have the same impact on reduced susceptibility to ATM-AVI and CAZ-AVI (Table 1).

### The combination of CMY-42 and the insertion of the four extra amino acids of PBP3 confers resistance to ATM-AVI.

To examine whether CMY-42 and PBP3 insertion together can confer ATM-AVI resistance, we introduced pET28a_CMY42 into 035125ΔpCMY42 by electroporation and introduced pET28a as a control. The MIC of ATM and ATM-AVI against strain 035125ΔpCMY42::pET28a_CMY42 was 512 and 32/4 mg/liter (Table 1), respectively. In contrast, the MIC of ATM and ATM-AVI against 035125ΔpCMY42::pET28a was 2 and 1/4 mg/liter, respectively. This confirms that *bla*_CMY-42_ and the PBP3 insertion in combination can confer ATM-AVI resistance. We also introduced pET28a_CMY2 into 035125ΔpCMY42 by electroporation to examine whether *bla*_CMY-2_ has the same effect as *bla*_CMY-42_. The MIC of ATM and ATM-AVI against 035125ΔpCMY42::pET28a_CMY2 was 32 and 1/4 mg/liter, respectively (Table 1). This indicates that, unlike *bla*_CMY-42_, *bla*_CMY-2_ cannot provide an additive effect to ATM-AVI resistance and to CAZ-AVI resistance (Table 1).

### The expression level of *bla*_CMY-42_ in the presence of the PBP3 amino acid insertion is correlated with the level of ATM-AVI resistance.

**FIG 2:**
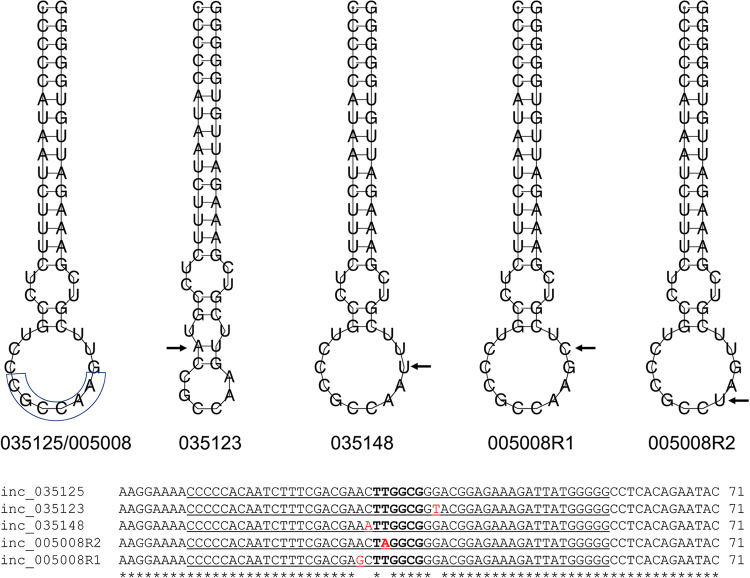
Alignments of the *inc* gene sequences and predicted RNA folding of Inc antisense RNA stem-loop region. (Top) Predicted RNA folding of Inc antisense RNA stem-loop region. The Inc RNA is transcribed from the complementary strand. The mutations are indicated by arrows. The hexanucleotides at the interaction sites with stem-loop 1 within RepZ mRNA are shown by a circle. (Bottom) Alignments of the *inc* gene sequences. The TTGGCG hexanucleotides, which are important sites for interaction between Inc RNA and stem-loop 1 within RepZ mRNA (23), are highlighted in boldface. The stem-loop region is underlined.

Compared to pCMY42_035125, there is a single SNP in the 71-bp *inc* gene on pCMY42_035123 (G38T) and another on pCMY42_035148 (C30A), the *bla*_CMY-42_-carrying plasmids of 035123 and 035148 (Fig. 2). To examine the expression of *bla*_CMY-42_ from the three plasmids, we introduced them into 035125ΔpCMY42 to construct 035125ΔpCMY42::pCMY42_035125, 035125ΔpCMY42::pCMY42_035123, and 035125ΔpCMY42::pCMY42_035148. We found that *bla*_CMY-42_ expression in 0035125ΔpCMY42::pCMY42_035123 and 0035125ΔpCMY42::pCMY42_035148 was increased 2.48- and 11.43-fold, respectively, compared to that of 035125ΔpCMY42::pCMY42_035125 by quantitative reverse transcription-PCR (qRT-PCR). We also determined the transcript level of *repZ*, which reflects the plasmid copy number (19), by qRT-PCR. The transcript levels of *repZ* of pCMY42_035123 and pCMY42_035148 were increased to 3.48- and 11.86-fold compared to that of pCMY42_035125, matching the levels observed for *bla*_CMY-42_ (Table 2 and Fig. S3). Correspondingly, the plasmid copy number of pCMY42_035123 and pCMY42_035148 was 7.00 and 26.81 per chromosome, respectively, and was higher than the value of 2.37 for pCMY42_035125 (Table 2).

**TABLE 2:** Expression level of *bla*_CMY-42_ and *repZ* (part of the IncIγ replicon) compared with strain 035125ΔpCMY42::pCMY42_035125

Strain::plasmid	Expression level (fold ± SD)		Plasmid copy no.^*a*^
	*bla*_CMY-42_	*repZ*	
035125ΔpCMY42::pCMY42_035123	2.48 ± 0.26	3.46 ± 1.22	7.00 ± 0.66
035125ΔpCMY42::pCMY42_035148	11.43 ± 2.06	11.86 ± 0.74	26.81 ± 1.91
035125ΔpCMY42::pCMY42_005008	0.64 ± 0.08	0.53 ± 0.03	2.50 ± 0.62
035125ΔpCMY42::pCMY42_005008R1	6.02 ± 1.68	6.49 ± 1.05	16.76 ± 3.11
035125ΔpCMY42::pCMY42_005008R2	6.02 ± 1.50	7.38 ± 2.20	14.84 ± 1.47

a The plasmid copy number refers to the ratio of pCMY42 plasmid copies per chromosome for each strain. The plasmid copy number of 035125ΔpCMY42::pCMY42_035125 is 2.37 ± 0.35.

10.1128/mSystems.00821-20.4 FIG S3 qRT-PCR results. (Left) Results for *bla*_CMY-42_. (Middle) Results for *repZ*. (Right) Results for *recA*. Download FIG S3, PDF file, 0.3 MB. Copyright © 2020 Ma et al. 2020 Ma et al. This content is distributed under the terms of the Creative Commons Attribution 4.0 International license.

### Two ATM-AVI-resistant mutants of strain 005008 had an SNP in *inc*.

**FIG 3:**
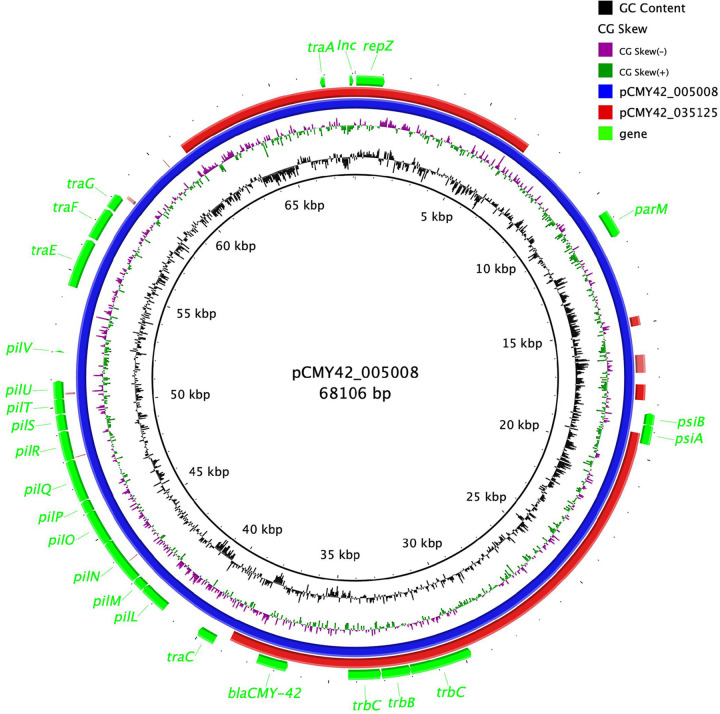
Comparison between pCMY42_005008 (68,106 bp) and pCMY42_035125 (34,321 bp). The figure was generated using BRIG (36), with GC skew and GC content being shown. Shown are *inc* and *repZ* of the IncI replicon, *bla*_CMY-42_. Compared with pCMY42_005008, pCMY42_035125 lacks a 20-kb conjugative region containing multiple *pil* genes (*pilL* to *pilV*) encoding the IncI thin pili (43) and several genes involved in the biogenesis of the IncI1 thick pili, such as *traE*, *traF*, and *traG* (44). pCMY42_005008 also lacks many *tra* genes encoding the IncI thick pili, such as *traI*, *traX*, and *traY* (44), compared with the well-characterized IncIγ reference plasmid R621a (GenBank accession no. AP011954). Other genes shown here include *psiA-psiB* (encoding plasmid SOS inhibition), *parM* (involved in plasmid partition), and *trb* genes (also involved in conjugative transfer).

Surprisingly, strain 005008 (17) had *bla*_CMY-42_ and the YRIN insertion in PBP3 but was susceptible to ATM-AVI (MIC, 4/4 mg/liter). To elucidate why, we obtained the complete sequence of the *bla*_CMY-42_-carrying plasmid (pCMY42_005008). pCMY42_005008 is a 68,106-bp IncIγ plasmid, significantly larger than the 34,321-bp pCMY42_035125 (Fig. 3), but the two plasmids have the same IncIγ replicon sequence. By qRT-PCR, we found that *bla*_CMY-42_ expression in 005008 was 0.64-fold higher than that in 035125 (Table 2), which could explain the discrepancy in ATM-AVI MIC. We then performed mutagenesis experiments for 005008 and obtained two ATM-AVI-resistant (MIC, 64/4 mg/liter; Table 1) mutants, 005008R1 and 005008R2. We performed genome sequencing for both and found that the two mutants were different from the parental strain by a single SNP at different positions in the IncIγ *inc* gene, A29G for 005008R1 and T32A for 005008R2 (Fig. 2), compared with the parental strain 005008. The *bla*_CMY-42_-carrying plasmids of 005008, 005008R1, and 005008R2 were introduced into 0035125ΔpCMY42 by electroporation. The presence of either A29G or T32A mutation led to a 16-fold increase in ATM-AVI MIC (64/4 versus 4/4 mg/liter; Table 1). qRT-PCR revealed that *bla*_CMY-42_ expression in 005008R1 and 05008R2 was 9.4-fold higher than that in 005008 and 6.0-fold higher than that in 035125ΔpCMY42::pCMY42_035125 (Table 2). Correspondingly, the *bla*_CMY-42_-carrying plasmid copy number increased from 2.50 per chromosome in 005008 to 16.76 in 005008R1 and 14.84 in 005008R2 (Table 2). Of note, the impact seen for CAZ-AVI was the same as that for ATM-AVI (Table 2).

## DISCUSSION

ATM mainly binds to PBP3 to achieve its antibacterial effect (20). The four-amino-acid (YRIK, YRIN, or YRIP) insertion is located in the tight turn between the β2b–β2c sheets adjacent to the β-lactam binding pocket (13). Such an insertion would disrupt the tight β-sheet and, therefore, would hinder the efficient binding of β-lactams such as ATM and CAZ (13). In a previous study (13), strains with the YRIK insertion have been shown to have a slightly higher ATM-AVI MIC (8 to 16 versus 4 mg/liter) than those with the YRIN insertion. However, such observations were based on strains with a different clonal background. Our analysis shows that the three types of amino acid insertion (YRIK, YRIN, and YRIP) have the same impact on ATM-AVI. This confirms that the location rather than the sequence of the insertion is critical to reduce the affinity to certain β-lactams. The amino acid insertions do not interfere with the essential transpeptidase function of PBP3 (13). Therefore, the insertion in PBP3 alone is inadequate to confer resistance to ATM-AVI.

In the presence of the PBP3 insertion, production of CMY-42 leads to ATM-AVI resistance. Compared to CMY-2, CMY-42 has enhanced activity against ATM. Although AVI can inhibit CMY-42, such inhibition appears to be compromised, as evidenced by the slightly increased ATM-AVI MIC against the strain producing CMY-42 compared to that producing CMY-2 (0.03/4 versus 0.015/4 mg/liter; Table 1) in the absence of the PBP3 insertion. A previous study has found that eight amino acids of AmpC cephalosporinases, i.e., Ser64, Lys67, Gln120, Tyr150, Asn152, Thr316, Lys315, and Asn346 (Ambler’s positions), are the key residues to interact with AVI (21). The amino acid substitution in CMY-42 is not one of the eight key residues and, therefore, is unlikely to significantly interfere with the inhibition by AVI. Another previous study exhibited that the amino acid substitution (Val231Gly) in CMY-30, which also occurs at the same position (Ambler’s position 211, part of the Ω loop) as CMY-42 (Val231Ser) compared to CMY-2, leads to enhanced hydrolysis against ATM and CAZ but not against carbapenems (22). This appears to be due to the more remote position of the R1 side chain of ATM and CAZ (both agents share the same R1 side chain in structure) from the amino acid at Ambler’s position 211, which therefore reduces the possibility for steric clashes (22). The additional polar bond between the hydrogen atom of 231Ser and oxygen atom of 232Ser in CMY-42 (Fig. S1) could reduce the interference of atoms at that position (231 from the start codon, Ambler’s position 211) and, therefore, may allow more interaction between the enzyme and the substrate, e.g., ATM and CAZ.

In the replicon of the I-complex family, such as IncI1 and IncIγ plasmids, the antisense regulatory Inc RNA encoded by *inc* folds into a single stem-loop (23) and binds to RepZ mRNA, transcripting the replication initiation protein RepZ (24). All mutations found in the *bla*_CMY-42_-carrying plasmids of 035125, 035148, 005008R1, and 005008R2 are located in the stem-loop region of *inc*. SNPs in the *inc* loop region can interfere with the binding of the antisense RNA and RepZ mRNA and, therefore, reduce the ability of *inc* to inhibit *repZ* translation, resulting in enhanced IncI plasmid replication and increased plasmid copy number (23, 25). Therefore, the expression of *bla*_CMY-42_ is enhanced in these IncIγ plasmids with *inc* mutations, as demonstrated in a previous study, in which the increased expression of *bla*_CMY-2_ in an IncI1 plasmid due to *inc* mutations led to resistance to piperacillin-tazobactam (19). The hexanucleotides TTGGCG (positions 31 to 36) in the *inc* loop region are interaction sites with RepZ mRNA (23, 25). The mutations seen in 035125 (G38T), 035148 (C30A), 005008R1 (A29G), and 005008R2 (T32A) are all located adjacent to or in the TTGGCG hexanucleotides (Fig. 2) but led to varied levels of reduced inhibition of *repZ*. This suggests that mutations in the loop region at different locations or with different nucleotides confer differential impacts on plasmid copy number control, which warrants further studies.

The reduced affinity of PBP3 to ATM increases the exposure of the agent to CMY-42, a class C β-lactamase. Unlike β-lactam-type β-lactamase inhibiters, such as clavulanic acid, sulbactam, or tazobactam, the inhibition of β-lactamases by AVI is reversible by recyclization (21, 26) and usually does not result in hydrolysis (21). This suggests that even in the presence of AVI, some free forms of CMY-42 enzymes still exist and, therefore, are available to attack ATM. CMY-42 has strong hydrolyzing activity against ATM, and the increased amount of the enzyme resulting from the increased copy number of the *bla*_CMY-42_-carrying plasmid would likely be sufficient to significantly reduce the amount of ATM to reach its target, PBP3, and then realizes resistance to ATM-AVI. This is also supported by the fact that an increase of AVI to 8 mg/liter largely restores the susceptibility of ATM, as shown in Table 1. It is worth pointing out that despite the present study focusing on ATM-AVI, the above-described mechanisms, including PBP3 insertion and the enhanced expression of *bla*_CMY-42_, have impacts on CAZ-AVI equal to those of ATM-AVI, as demonstrated in Results.

In conclusion, the concerted combinatorial effect of three elements, i.e., target alteration, hydrolyzing enzyme, and plasmid expression, is able to overcome the protection of AVI for ATM and CAZ, leading to clinically relevant resistance in E. coli.

## MATERIALS AND METHODS

### Strains and *in vitro* susceptibility testing.

E. coli isolates 035125, 035123, and 035148 (all of sequence type 410 [ST410]) were recovered from hospital sewage of West China Hospital in March 2018. Carbapenem-resistant E. coli (CREC) clinical strain 005008 (ST3835) was recovered from ascites of an intensive care unit patient in 2014 (17), while CREC strain 020066 (ST6823) was recovered from urine of a hospitalized patient in 2017 (18).

A 200-μl sample of hospital sewage was collected from the influx mainstream of the wastewater treatment plant at West China Hospital in November 2017 and then was streaked onto a chromogenic agar plate (CHROMagar enterobacteria; CHROMagar; Paris, France) containing 8/4 mg/liter ATM-AVI and 64 mg/liter linezolid. The addition of linezolid was to inhibit the growth of Gram-positive bacteria. The plate was then incubated at 37°C overnight. The colonies were picked and streaked on the same types of plates as those described above for purification. Preliminary species identification was based on the matrix-assisted laser desorption ionization time-of-flight mass spectrum (MALDI-TOF) (Bruker; Billerica, MA).

MICs of amikacin, ATM, ATM-AVI, CAZ, CAZ-AVI, ciprofloxacin, colistin, imipenem, meropenem, piperacillin-tazobactam, tigecycline, and trimethoprim-sulfamethoxazole were determined using the broth microdilution method of the Clinical and Laboratory Standards Institute (CLSI) (27). In addition, to test whether increased concentrations of AVI could enhance the protection for ATM and CAZ, MICs of ATM and CAZ were also determined in the presence of 8 mg/liter AVI. The breakpoints of ATM defined by the CLSI were applied for ATM-AVI and ATM-AVI (8 mg/liter AVI), and those of CAZ-AVI were also applied for CAZ-AVI (8 mg/liter AVI). Of note, throughout the manuscript, AVI in ATM-AVI and CAZ-AVI is at 4 mg/liter unless indicated otherwise. As there are no breakpoints of tigecycline from the CLSI, those defined by EUCAST (http://www.eucast.org/) were applied.

### Whole-genome sequencing and analysis.

Genomic DNA of 035125, 035123, 035148, 005008, 005008R1, and 005008R2 was extracted using the QIAamp DNA minikit (Qiagen, Hilden, Germany), and whole-genome sequencing was performed using a HiSeq X10 platform (Illumina, San Diego, CA, USA). Sequence reads were subjected to strict quality control using Cutadapt v2.5 (28) and BBTools v38.68 (https://sourceforge.net/projects/bbmap/) until no further improvements were observed on the reads. Trimmed reads were downsampled to 100× depth if exceeding this threshold and then assembled into draft genomes with a minimum contig size of 200 bp using SPAdes v3.14.1 (29) invoked in Shovill v1.0.9 (https://github.com/tseemann/shovill) under careful mode.

035125, 035123, 035148, and 005008 were also sequenced using a long-read MinION sequencer (Nanopore, Oxford, UK). Genomic DNA was prepared using phenol-chloroform to minimize fragmented DNA. The output long reads were base called and demultiplexed using Guppy v3.2.4 (https://nanoporetech.com/nanopore-sequencing-data-analysis). Short Illumina reads and long MinION reads were subjected to *de novo* hybrid assembly using Unicycler v0.4.8 (30) under the conservative mode for improving accuracy. Complete circular contigs were then corrected and polished using Pilon v1.22 (31), in addition to the integrated polishing steps in Unicycle. A quality check was performed on the assembled genomes using CheckM v1.0.18 (32) to determine the existence of contamination. Genomes were annotated using Prokka v1.14.5 (33). ST was determined by querying the multilocus sequence typing database of E. coli (http://enterobase.warwick.ac.uk/species/index/ecoli). Antimicrobial resistance genes were predicted using AMRFinderPlus v3.2.3 (34). Plasmid replicons were identified using ABRicate v.0.9.8 (https://github.com/tseemann/abricate) with PlasmidFinder (35). The comparison between the *bla*_CMY-42_-carrying plasmid of 035125 and that of 005008 was performed using BRIG (36) with default settings.

Reads of 035123, 035125, and 035148 were aligned using Snippy v4.4.5 (https://github.com/tseemann/snippy) with default settings. Recombination was detected using Gubbins v2.3.4 (37) with a maximum of 100 iterations for convergence under the GTRGAMMA model. A pair-wised core SNP distance excluding SNPs residing in the recombination regions was calculated using snp-dists v0.6.3 (https://github.com/tseemann/snp-dists). The same procedure was also used for SNP calling between 005008 and its mutants.

### Curation of plasmids by SDS treatment.

To obtain a *bla*_CMY-42_-carrying plasmid-cured variant of strain 035125, the strain was incubated in 10 ml fresh Luria-Bertani (LB) broth containing 0.02% SDS at 37°C overnight (38). The culture was diluted 10^−6^ in fresh LB broth, and a 100-μl aliquot was plated onto an LB agar plate. The bacterial colonies were transferred using sterilized sticks onto LB agar plates with and without 16 mg/liter ATM simultaneously. The procedure was repeated until the strain lost resistance to ATM, indicating the curation of plasmid pCMY42_035125. The absence of *bla*_CMY-42_ and the IncIγ replicon from susceptible colonies was confirmed by PCR using self-designed primers IncI-R/L and cmy42-R/L (Table 1). The *bla*_CMY-42_-carrying plasmid-cured variant of strain 035125 was assigned the name 035125ΔpCMY42.

### Cloning.

The penicillin binding protein 3 (PBP3)-encoding gene *pbp3* of 035125, 005008, and 020066 was cloned to examine the impact of the four-amino-acid insertion on ATM-AVI resistance, with *pbp3* of E. coli BL21 being cloned as a control. The *pbp3* complete sequences of 035125, 005008, 020066, and BL21 were amplified using PCR with PrimeSTAR Max DNA polymerase (primers are listed in Table 3; TaKaRa; Dalian, China). PCR amplicons and the pBC SK vector (Stratagene, La Jolla, CA, USA) were digested using BamHI and EcoRI (New England Biolabs, Ipswich, MA, USA) and were then ligated using T4 ligase (New England Biolabs) to construct pBC SK-*pbp3*_YPIK, pBC SK-*pbp3*_YPIN, pBC SK-*pbp3*_YPIP, and pBC SK-*pbp3*_BL21, which was transformed into E. coli BL21 by chemical transformation. Potential transformants were selected on LB agar plates containing 50 mg/liter chloramphenicol. The presence of the cloned fragments was confirmed by PCR using generic primers M13-20/M13 with reverse binding to the cloning region of pBC SK and subsequent Sanger sequencing.

**TABLE 3:** Primers used in this study

Primer	Sequence^*a*^ (5′–3′)	Size (bp)	Target gene or region
pCMY42-IncI-R	GCATTCAGGAGAGATGGCAT	141	IncI replicon
pCMY42-IncI-L	CCCGCCAAGTTCGTCGAAAG		
pCMY42-cmy42-R	CTGGGAGATGCTGAACTGGC	148	*bla*_CMY-42_
pCMY42-cmy42-L	AGTGGAGCCCGTTTTATGCA		
pET-IS1-Nhel-up	AATGCTAGCCAACACGATTTTCCGCCATT	2,100	*bla*_CMY-42_
pET-cmy42-BamHI-dw	AAAGGATCCAAAGGAGGCCCAATATCCTG		
pET-ISEcp1-Nhel-up	AAAGCTAGCCACTGCAAACGGTGCTGCGG	2,218	*bla*_CMY-2_
pET-cmy2-BamHI-dw	AAAGGATCCAAAGGAGGCCCAATATCCTG		
pBCSK-pbp3-EcoRI-up	AGCGAATTCATGAAAGCAGCGGCGAAAAC	1,779	*pbp3*
pBCSK-pbp3-BamHI-dw	CAAGGATCCCGGTTACGATCTGCCACCTGTCCC		
cmy42/cmy2-qpcr-R	TCGCCAATAACCACCCAGTC	125	*bla*_CMY-42_/*bla*_CMY-2_
cmy42/cmy2-qpcr-L	GACCGGATCGCTGAGCTTAA		
repZ-qpcr-R	CTGGAGTCAGTTAGCACCCG	140	*repZ*
repZ-qpcr-L	CGCATTTGGGTTTGGTGGAG		
recA-qpcr-L	GTAAAGGCTCCATCATGCGC	135	*recA*
recA-qpcr-R	AGATTTCGACGATACGGCCC		

a Restriction sites are underlined.

The −10 and −35 boxes of the promoter of *bla*_CMY-2_ and *bla*_CMY-42_ were predicted using the online tool BPROM (http://www.softberry.com/). The complete coding sequences of *bla*_CMY-2_ and *bla*_CMY-42_ and their promoter regions were amplified from strains 020147 (E. coli ST410) (18) and 035125, respectively, using PrimeSTAR Max DNA polymerase (primers are in Table 3). PCR amplicons and the pET-28a vector (Fenghui, Changsha, China) were digested using BamHI and NheI and then were ligated using T4 ligase to construct pET28a_CMY42 and pET28a_CMY2, which were transformed into E. coli BL21 by chemical transformation. Potential transformants were selected on LB agar plates containing 50 mg/liter amikacin. The presence of *bla*_CMY-2_ or *bla*_CMY-42_ in transformants was confirmed by PCR with the same primers for cloning and subsequent Sanger sequencing of amplicons. MICs of ATM, ATM-AVI, CAZ, and CAZ-AVI were determined for the aforementioned transformants using the CLSI broth microdilution method (27).

### Conjugation and electroporation experiments.

Conjugation experiments were carried out in broth and on filters with the azide-resistant E. coli strain J53 AizR as the recipient at both 25 and 37°C as described previously (39). Potential transconjugants were selected on LB agar plates containing 16 mg/liter ATM. Plasmids were prepared using alkaline lysis (40). Electroporation was performed using the protocol for E. coli (41) using a Gene Pulser (Bio-Rad, Hercules, CA, USA). Transformants were selected on LB agar plates containing 16 mg/liter ATM, and the presence of *bla*_CMY-42_ in transformants was confirmed by PCR and subsequent Sanger sequencing of amplicons.

### qRT-PCR.

Overnight cultures of 035125, 035123, 035148, 005008, 005008R1, and 005008R2 were inoculated (1:100 dilution) into LB medium and were incubated at 37°C with vigorous shaking. At an optical density at 600 nm of 0.6, cell pellets were harvested by centrifugation, and RNA was extracted using the bacterial RNA kit (Omega Bio-Tek, Norcross, GA) with the treatment of on-membrane DNase I digestion to remove DNA contamination. Reverse transcription of RNA to cDNA was performed using a PrimeScrip RT reagent kit (TaKaRa). Gene-specific primers were designed using the Primer3 software (http://frodo.wi.mit.edu/). qRT-PCR of *bla*_CMY-42_ and *repZ* were carried out using LightCycler 96 (Roche, Basel, Switzerland) with FastStart essential DNA green master (Roche) and self-designed primers (Table 3). The housekeeping gene *recA* was used as an internal control for the quantification of relative gene expression. For each strain, three independent cultures were used to extract RNA as three biological replicates, and for each RNA sample, the whole process of qRT-PCR was repeated in triplicate as technical replicates. Relative transcript levels were calculated using the 2^−ΔΔCT^ formula based on the mean values.

### *In vitro* resistance mutation assay.

Strain 005008 was consecutively streaked on LB agar plates containing incremental concentrations of ATM-AVI from 2/4 to 32/4 mg/liter to obtain resistance mutants. Two such mutants, 005008R1 and 005008R2, were subjected to whole-genome sequencing using the HiSeq X10 platform as described above.

### RNA folding predictions.

Inc RNA folding was predicted using RNAfold WebServerRNA (http://rna.tbi.univie.ac.at/cgi-bin/RNAWebSuite/RNAfold.cgi).

### Protein structure predictions.

Protein structures of CMY-2 and CMY-42 were predicted using the tool I-TASSER (Iterative Threading ASSEmbly Refinement; https://zhanglab.ccmb.med.umich.edu/I-TASSER/). The molecular visualization tool PyMOL (https://pymol.en.softonic.com/) was used to display and analyze the diversity of protein residues.

### Plasmid copy number determination.

The plasmid copy numbers of pCMY42_035125, pCMY42_035123, pCMY42_035148, pCMY42_005008, pCMY42_005008R1, and pCMY42_005008R2 were determined after introduction into strain 035125ΔpCMY42 (the *bla*_CMY-42_-carrying, plasmid-cured variant of strain 035125) by real-time PCR. Genomic DNA of the corresponding strains was prepared using the QIAamp DNA minikit (Qiagen). Real-time PCR was performed using LightCycler 96 (Roche) with FastStart essential DNA green master (Roche) and self-designed primers (Table 3) for *repZ* and the housekeeping gene *recA*. The whole process of real-time PCR was repeated in triplicate. Relative plasmid copy numbers were calculated using the 2^−ΔΔCT^ formula for *repZ* compared with that for *recA* based on the mean values.

### Data availability.

Complete genomes of strains 035125, 035123, 035148, and 005008 have been deposited in GenBank under accession numbers CP029365 to CP029369 and CP058651 to CP058665. Draft genomes of 005008R1 and 005008R2 have been deposited in GenBank under the accession numbers JACCKG000000000 and JACCKF000000000, respectively.
