# Supplementary material for: Struggle To Survive: the Choir of Target Alteration, Hydrolyzing Enzyme, and Plasmid Expression as a Novel Aztreonam-Avibactam Resistance Mechanism
Source: mSystems. 2020 Nov 3;5(6):e00821-20. doi: 10.1128/mSystems.00821-20 (PMC7646527; doi:10.1128/mSystems.00821-20)
Supplement: TABLE S1 [file mSystems.00821-20-st001.docx]

Table S1. MICs (mg/L) of antimicrobial agents against strains 035123, 035125, 035148, 005008 and 005008R1.

|  | 035125 | 035123 | 035148 | 005008 | 005008R1 | 005008R2 |
| --- | --- | --- | --- | --- | --- | --- |
| Aztreonam | **128** | **512** | **512** | **>512** | **>512** | **>512** |
| Aztreonam-avibactam (4 mg/L) | **16/4** | **64/4** | **128/4** | 4/4 | **64/4** | **64/4** |
| Aztreonam-avibactam (8 mg/L) | 1/8 | **16/8** | **32/8** | 0.5/8 | 4/8 | 4/8 |
| Ceftazidime | **1,024** | **>1,024** | **>1,024** | **>1,024** | **>1,024** | **>1,024** |
| Ceftazidime-avibactam (4 mg/L) | 8/4 | **16/4** | **32/4** | **64/4** | **256/4** | **256/4** |
| Ceftazidime-avibactam (8 mg/L) | 0.5/8 | 1/8 | 4/8 | **16/8** | **32/8** | **32/8** |
| Piperacillin-tazobactam | **1,024/4** | **>1,024/4** | **>1,024/4** | **>1,024/4** | **>1,024/4** | **>1,024/4** |
| Meropenem | 0.015 | 0.03 | 0.03 | **128** | **128** | **128** |
| Imipenem | 0.06 | 0.125 | 0.125 | **>256** | **>256** | **>256** |
| Amikacin | 4 | 4 | 4 | 4 | 4 | 4 |
| Ciprofloxacin | **512** | **512** | **512** | **512** | **512** | **512** |
| Colistin | 0.5 | 0.5 | 0.5 | 0.5 | 0.5 | 0.5 |
| Tigecycline | ≤0.5 | ≤0.5 | ≤0.5 | 0.5 | 0.5 | 0.5 |
| Trimethoprim-sulfamethoxazole | **128/**  **2,432** | **128/**  **2,432** | **128/**  **2,432** | **128/**  **2,432** | **128/**  **2,432** | **128/**  **2,432** |

Strain 005008R1 and 005008R2 are mutants of 005008.

Susceptibility category: resistance is highlighted in bold and intermediate is underlined.
