# Supplementary material for: Struggle To Survive: the Choir of Target Alteration, Hydrolyzing Enzyme, and Plasmid Expression as a Novel Aztreonam-Avibactam Resistance Mechanism
Source: mSystems. 2020 Nov 3;5(6):e00821-20. doi: 10.1128/mSystems.00821-20 (PMC7646527; doi:10.1128/mSystems.00821-20)
Supplement: FIG S2 [file mSystems.00821-20-sf002.pdf]

```
005008      MKAAAKTQKPKRQEEHANFISWRFALLCGCILLALAFLLGRVAWLQVISPDMLVKEGDMR  60
035123      MKAAAKTQKPKRQEEHANFISWRFALLCGCILLALAFLLGRVAWLQVISPDMLVKEGDMR  60
035125      MKAAAKTQKPKRQEEHANFISWRFALLCGCILLALAFLLGRVAWLQVISPDMLVKEGDMR  60
035148      MKAAAKTQKPKRQEEHANFISWRFALLCGCILLALAFLLGRVAWLQVISPDMLVKEGDMR  60
020066      MKAAAKTQKPKRQEEHANFISWRFALLCGCILLALAFLLGRVAWLQVISPDMLVKEGDMR  60
BL21de3     MKAAAKTQKPKRQEEHANFISWRFALLCGCILLALAFLLGRVAWLQVISPDMLVKEGDMR  60
            ************************************************************

005008      SLRVQQVSTSRGMITDRSGRPLAVSVPVKAIWADPKEVHDAGGISVGDRWKALANALNIP  120
035123      SLRVQQVSTSRGMITDRSGRPLAVSVPVKAIWADPKEVHDAGGISVGDRWKALANALNIP  120
035125      SLRVQQVSTSRGMITDRSGRPLAVSVPVKAIWADPKEVHDAGGISVGDRWKALANALNIP  120
035148      SLRVQQVSTSRGMITDRSGRPLAVSVPVKAIWADPKEVHDAGGISVGDRWKALANALNIP  120
020066      SLRVQQVSTSRGMITDRSGRPLAVSVPVKAIWADPKEVHDAGGISVGDRWKALANALNIP  120
BL21de3     SLRVQQVSTSRGMITDRSGRPLAVSVPVKAIWADPKEVHDAGGISVGDRWKALANALNIP  120
            ************************************************************

005008      LDQLSARINANPKGRFIYLARQVNPDMADYIKKLKLPGIHLREESRRYYPSGEVTAHLIG  180
035123      LDQLSARINANPKGRFIYLARQVNPDMADYIKKLKLPGIHLREESRRYYPSGEVTAHLIG  180
035125      LDQLSARINANPKGRFIYLARQVNPDMADYIKKLKLPGIHLREESRRYYPSGEVTAHLIG  180
035148      LDQLSARINANPKGRFIYLARQVNPDMADYIKKLKLPGIHLREESRRYYPSGEVTAHLIG  180
020066      LDQLSARINANPKGRFIYLARQVNPDMADYIKKLKLPGIHLREESRRYYPSGEVTAHLIG  180
BL21de3     LDQLSARINANPKGRFIYLARQVNPDMADYIKKLKLPGIHLREESRRYYPSGEVTAHLIG  180
            ************************************************************

005008      FTNVDSQGIEGVEKSFDKWLTGQPGERIVRKDRYGRVIEDISSTDSQAAHNLALSIDERL  240
035123      FTNVDSQGIEGVEKSFDKWLTGQPGERIVRKDRYGRVIEDISSTDSQAAHNLALSIDERL  240
035125      FTNVDSQGIEGVEKSFDKWLTGQPGERIVRKDRYGRVIEDISSTDSQAAHNLALSIDERL  240
035148      FTNVDSQGIEGVEKSFDKWLTGQPGERIVRKDRYGRVIEDISSTDSQAAHNLALSIDERL  240
020066      FTNVDSQGIEGVEKSFDKWLTGQPGERIVRKDRYGRVIEDISSTDSQAAHNLALSIDERL  240
BL21de3     FTNVDSQGIEGVEKSFDKWLTGQPGERIVRKDRYGRVIEDISSTDSQAAHNLALSIDERL  240
            ************************************************************

005008      QALVYRELNNAVAFNKAESGSAVLVDVNTGEVLAMANSPSYNPNNLSGTPKEAMRNRTIT  300
035123      QALVYRELNNAVAFNKAESGSAVLVDVNTGEVLAMANSPSYNPNNLSGTPKEAMRNRTIT  300
035125      QALVYRELNNAVAFNKAESGSAVLVDVNTGEVLAMANSPSYNPNNLSGTPKEAMRNRTIT  300
035148      QALVYRELNNAVAFNKAESGSAVLVDVNTGEVLAMANSPSYNPNNLSGTPKEAMRNRTIT  300
020066      QALVYRELNNAVAFNKAESGSAVLVDVNTGEVLAMANSPSYNPNNLSGTPKEAMRNRTIT  300
BL21de3     QALVYRELNNAVAFNKAESGSAVLVDVNTGEVLAMANSPSYNPNNLSGTPKEAMRNRTIT  300
            ************************************************************

005008      DVFEPGSTVKPMVVMTALQRGVVRENSVLNTIPYRINYRINGHEIKDVARYSELTLTGVL  360
035123      DVFEPGSTVKPMVVMTALQRGVVRENSVLNTIPYRIKYRINGHEIKDVARYSELTLTGVL  360
035125      DVFEPGSTVKPMVVMTALQRGVVRENSVLNTIPYRIKYRINGHEIKDVARYSELTLTGVL  360
035148      DVFEPGSTVKPMVVMTALQRGVVRENSVLNTIPYRIKYRINGHEIKDVARYSELTLTGVL  360
020066      DVFEPGSTVKPMVVMTALQRGVVRENSVLNTIPYRIPYRINGHEIKDVARYSELTLTGVL  360
BL21de3     DVFEPGSTVKPMVVMTALQRGVVRENSVLNTIP----YRINGHEIKDVARYSELTLTGVL  356
            *********************************    ***********************
```
