# Supplementary figures and images for: Struggle To Survive: the Choir of Target Alteration, Hydrolyzing Enzyme, and Plasmid Expression as a Novel Aztreonam-Avibactam Resistance Mechanism
Source: mSystems. 2020 Nov 3;5(6):e00821-20. doi: 10.1128/mSystems.00821-20 (PMC7646527; doi:10.1128/mSystems.00821-20)

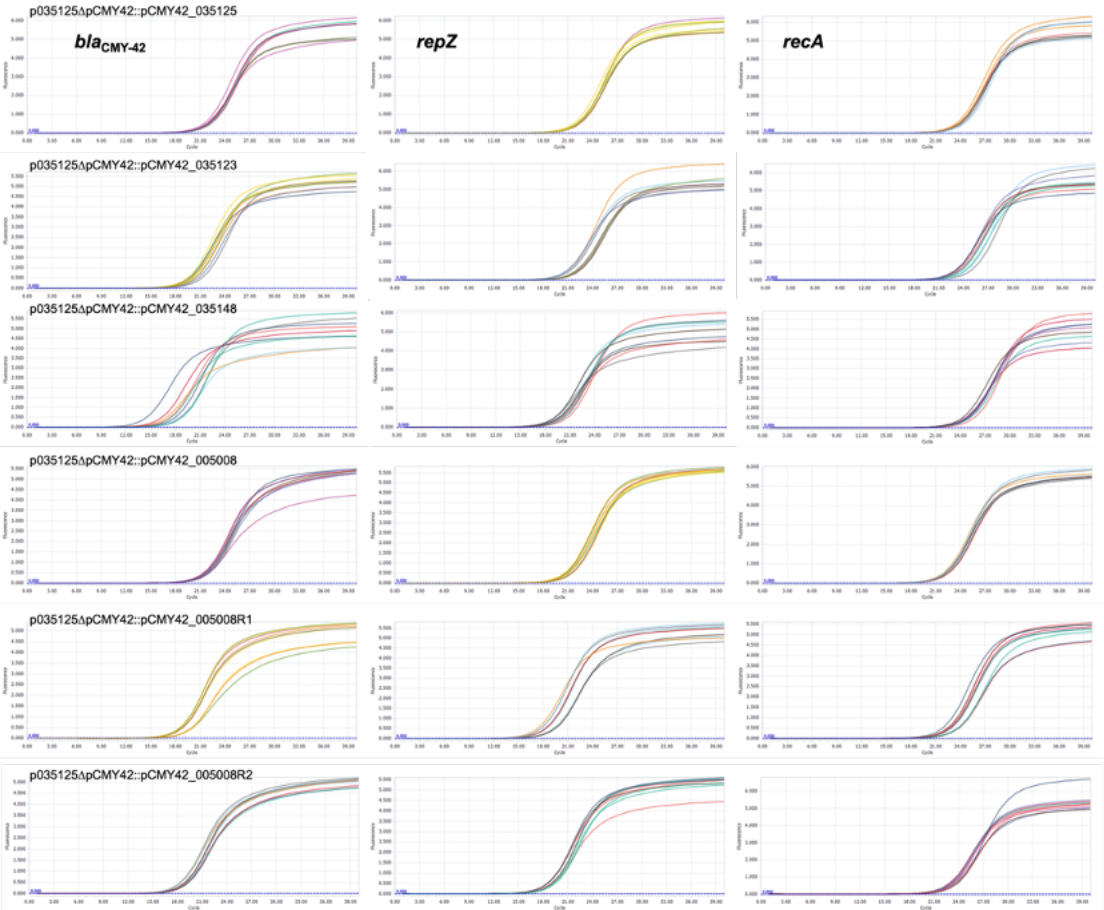

Supplement: FIG S3 [file mSystems.00821-20-sf003.pdf]
