# Supplementary material for: Struggle To Survive: the Choir of Target Alteration, Hydrolyzing Enzyme, and Plasmid Expression as a Novel Aztreonam-Avibactam Resistance Mechanism
Source: mSystems. 2020 Nov 3;5(6):e00821-20. doi: 10.1128/mSystems.00821-20 (PMC7646527; doi:10.1128/mSystems.00821-20)
Supplement: FIG S1 [file mSystems.00821-20-sf001.pdf]

A

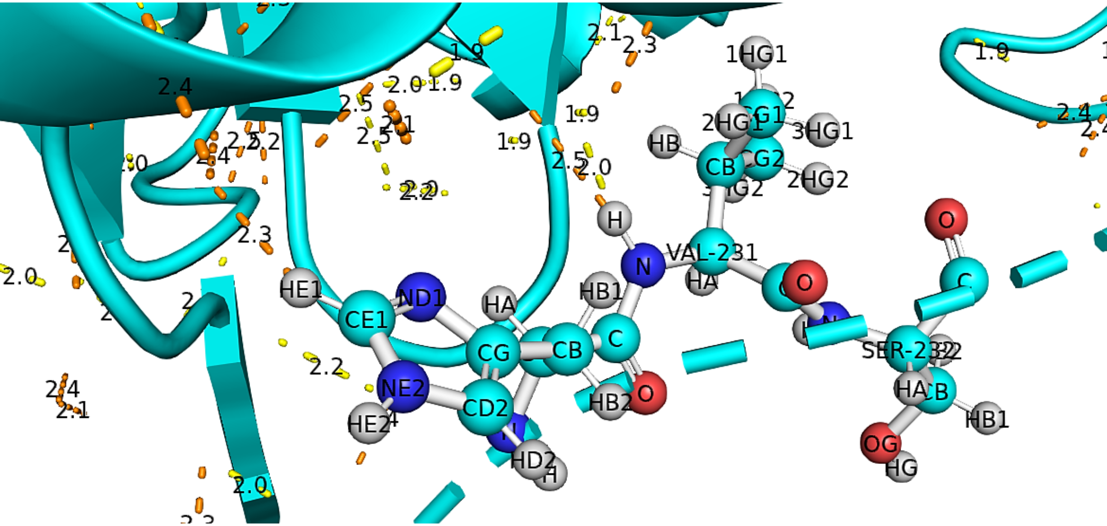
VAL-231
SER-232
HE1
CE1
ND1
HA
CG
CB
C
HB1
N
H
HA
NE2
CD2
HE2
HD2
HB2
O
HB
CB
1HG1
3HG1
2HG1
2HG2
O
C
HA
CB
HB1
OG
HG

B

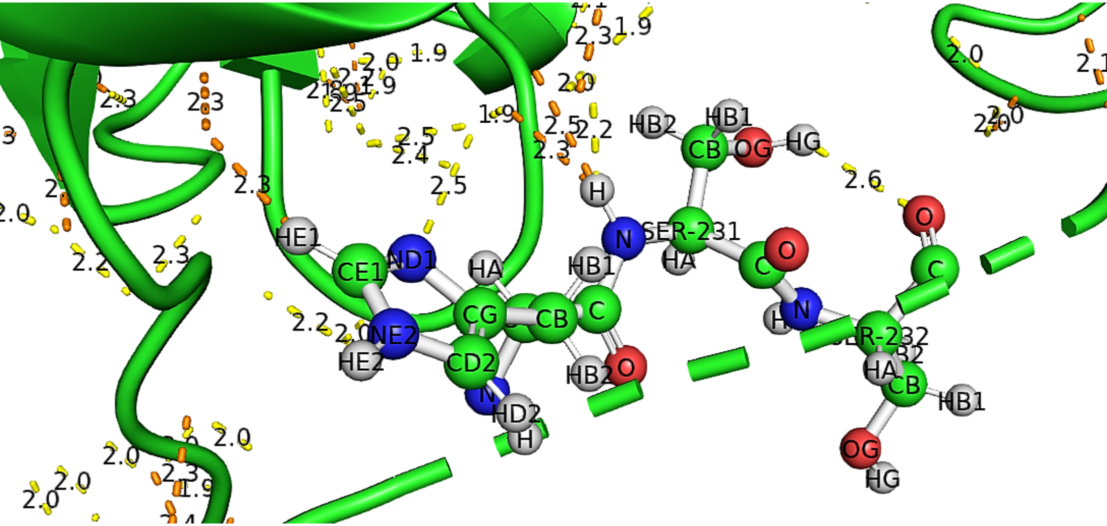
SER-231
HE1
CE1
ND1
HA
CG
CB
C
HB1
N
H
HA
HB2
HB1
CB
OG
HG
2.6
O
C
N
NE2
HE2
CD2
HD2
HB2
O
HA
CB
HB1
OG
HG
